# Weight Trajectory Since Birth, Current Body Composition, Dietary Intake, and Glucose Tolerance in Young Underweight Japanese Women

**DOI:** 10.1089/whr.2021.0127

**Published:** 2022-02-14

**Authors:** Mika Takeuchi, Mari Honda, Ayaka Tsuboi, Satomi Minato-Inokawa, Miki Kurata, Bin Wu, Tsutomu Kazumi, Keisuke Fukuo

**Affiliations:** ^1^ Research Institute for Nutrition Sciences, Mukogawa Women's University, Nishinomiya, Hyogo, Japan.; ^2^ Open Research Center for Studying of Lifestyle-Related Diseases, Mukogawa Women's University, Nishinomiya, Hyogo, Japan.; ^3^ Department of Health, Sports, and Nutrition, Faculty of Health and Welfare, Kobe Women's University, Kobe, Hyogo, Japan.; ^4^ Department of Nutrition, Osaka City Juso Hospital, Osaka, Japan.; ^5^ Laboratory of Community Health and Nutrition, Department of Bioscience, Graduate School of Agriculture, Ehime University, Matsuyama, Ehime, Japan.; ^6^ Department of Food Sciences and Nutrition, School of Food Sciences and Nutrition, Mukogawa Women's University, Nishinomiya, Hyogo, Japan.; ^7^ Department of Endocrinology, First Affiliated Hospital of Kunming Medical University, Kunming, Yunnan, China.; ^8^ Department of Medicine, Kohnan Kakogawa Hospital, Kakogawa, Hyogo, Japan.

**Keywords:** underweight, weight history, body composition, young Japanese women

## Abstract

***Introduction:*** We studied weight trajectory since birth and dietary intake in Japanese female students majoring in nutrition sciences.

***Materials and Methods:*** Birth weight, adolescent height and weight, current body composition by whole-body dual-energy X-ray absorptiometry, dietary intake, glucose tolerance, lipid profile, and adipokines were cross-sectionally compared between young underweight (body mass index [BMI] <18.5) and normal-weight (BMI ≥18.5 and <25.0) women with overweight (BMI ≥25.0) women as an internal reference.

***Results:*** Serum adiponectin (leptin) was the highest (lowest) in 42 underweight women, intermediate levels in 251 normal-weight women, and the lowest (highest) levels in 14 overweight women. Compared with normal-weight women, underweight women had lower weight at birth, at age 12, 15, and 20 years, but comparable height, and hence lower BMI at three time points. Underweight women had higher gluteofemoral fat adjusted for total body fat and weight-adjusted skeletal muscle mass, although absolute and height-adjusted fat mass and skeletal muscle mass were lower. Glucose tolerance assessed by oral glucose testing, serum triglycerides, and high-density lipoprotein cholesterol did not differ between the two groups. Daily intake expressed per kg of body mass of energy and protein was the highest in underweight women, intermediate levels in normal-weight women, and the lowest levels in overweight women.

***Conclusions:*** Some young Japanese women are underweight not because of a strong drive for thinness, but because they were born lighter and remained lean until young adults. Underweight was associated with higher gluteofemoral fat adjusted for total body fat and relative skeletal muscle mass.

## Introduction

Obesity has become a major health problem worldwide. Mean body mass index (BMI) was on the rise over time in Japanese men and older women.^1,2^ In contrast, BMI was on the decline in younger Japanese women.^1–4^ As a consequence, the prevalence of underweight or thinness (BMI <18.5 kg/m^2^) in young women increased from 15.8% in 1976–1980 to 22.9% in 1996–2000^3^ and still remained high (21.7%) in 2016.^1^ The thinness prevalence rate in young women was higher in Japan compared to other countries. For example, it was 11.6% in Australian women 18–23 years of age (1996),^5^ and 11.4% (2000), 17.1% (2010), and 12.9% (2017) in female 1st-year university students in Lithuania.^6^

A higher thinness prevalence rate would be counterintuitive in the current obesity era. Although reasons of the increase in thinness in young Japanese women remain unclear, studies found a negative body image and weight-loss behaviors among a substantial proportion of normal-weight children and adolescents.^7–9^ This could manifest as a rising thinness prevalence. Indeed, some have suggested that programs to prevent overweight and obesity may actually cause unintended harm.^10,11^

It has recently demonstrated that the prevalence of presarcopenia (low muscle mass) was significantly elevated among the age group of 18–39 years from 11.3% in 1999–2000 to 14.1% in 2005–2006.^12^ Since skeletal muscle is a major site of insulin-mediated glucose disposal in the postprandial state,^13^ its decline could be associated with insulin resistance. Skeletal muscle mass can be evaluated by weight-adjusted relative skeletal muscle as the percentage of body mass and height-adjusted skeletal muscle mass. Although height-adjusted skeletal muscle mass is used in the diagnosis of sarcopenia,^14^ it has been shown that relative muscle mass is related inversely to insulin resistance, whereas absolute muscle mass is related positively.^15,16^ Studies found that low relative muscle mass was associated with an increased risk of type 2 diabetes independent of general obesity.^17,18^

To the best of our knowledge, studies are missing on weight trajectories since birth in underweight young women. In addition, studies are limited on detailed body composition and glucose tolerance in this population. In this study, we studied weight trajectory since birth and dietary intake in Japanese female students majoring in nutrition sciences because reasons of high prevalence of thinness in young Japanese women remain unclear, and because students majoring in nutrition sciences may be less likely to have unnecessary weight control.

## Methods

For this open research for studying lifestyle-related diseases, briefing sessions on the study were held once or twice every year between 2004 and 2007 for female collegiate athletes (Department of Health and Sports Sciences), as well as students of Department of Food Sciences and Nutrition (nonathletes), Mukogawa Women's University. Written leaflets, which included aims, design, and exclusion criteria described below, were provided in each session. At the end of every year, participants were invited to a meeting, where study results were explained. In 2004, body composition, dietary intake, and cardiometabolic features were compared between female collegiate athletes and nonathlete students.

In 2005, similar study was done in nonathlete students and their biological mothers. In 2006 and 2007, a standard 75 g oral glucose tolerance test (OGTT), in addition to similar measurements done in 2004, was performed in two groups of students and their parents. Meal tests were also done in two groups of students in 2007. Although the number of nonathlete students totaled 318, who participated between 2004 and 2007, 7 students underwent both fasting blood sampling in 2004 and OGTT. Their data on OGTT were included in analyses and their fasting data were not included. Thereafter, data on 311 nonathlete students were used for all analyses.

Among 311 students,^19,20^ 307 students^21,22^ whose genomic DNA was available were cross-sectionally studied in this study. They were recruited as volunteers. Subjects with clinically diagnosed acute or chronic inflammatory diseases, endocrine, cardiovascular, hepatic, or renal diseases, hormonal contraception, and unusual dietary habits were excluded. Nobody reported receiving any medication or having regular supplements. The study was approved by the Ethics Committees of the University (No. 07-28 on February 19, 2008) to be in accordance with the Helsinki declaration. All subjects gave written consent after the experimental procedure had been explained.

Among 307 women, 226 and 198 women provided weight data since birth and dietary intake of the previous month, respectively. Weight at birth, and height and weight at age 12 and 15 were obtained either through maternal health check notes or child health notebook records (issued by each municipal office). Dietary intake was assessed using the self-administered diet history questionnaire.^23^ This has been widely used throughout Japan and its validity with respect to commonly studied nutrition factors has been confirmed. There was no significant difference in anthropometric and biochemical measurements between women who provided and those who did not provide data on weight trajectories and dietary intake (data not shown).

After a 12-hour overnight fast at 8:30 AM, participants underwent blood sampling and measurements of anthropometric indices and body composition as previously described.^19,20^ They were divided into three groups: underweight (BMI <18.5 kg/m^2^), normal weight (BMI ≥18.5 kg/m^2^ and <25.0 kg/m^2^), and overweight (BMI ≥25.0 kg/m^2^). A subsample of 129 women underwent a standard 75 g OGTT. Blood was drawn at 0 (fasting), 30 minutes, 1 hour, and 2 hours for glucose and insulin measurements. In fasting blood samples, the following were also measured as previously reported: serum cholesterol, triglyceride, high-density lipoprotein (HDL) cholesterol, glycated hemoglobin (HbA1c), adiponectin, leptin, and high-sensitivity C-reactive protein (hsCRP).^19,20^

Plasma glucose was determined by the hexokinase/glucose-6-phosphate dehydrogenase method (interassay coefficient of variation [CV] <2%). Serum insulin was measured by an enzyme-linked immunosorbent assay method with a narrow specificity, excluding des-31, des-32, and intact proinsulin (inter-assay CV <6%). Impaired glucose tolerance (IGT) was defined as 2-hour glucose: 140–199 mg/dL.^24^ Insulin resistance and secretion were evaluated using homeostasis model assessment of insulin resistance (HOMA-IR) and secretion (HOMA-β), respectively.^25^

Fat mass, bone mass, and lean mass for arms, legs, trunk, and the total body were measured using whole-body dual-energy X-ray absorptiometry (DXA: Hologic QDR-2000, software version 7.20D, Waltham, MA) as previously reported.^21^ Muscle characteristics were relative lean mass (total body or appendicular lean mass [ALM] as percentage of body mass, %TBLM, and %ALM, respectively) and absolute lean mass (ALM/height^2^ in kg/m^2^ [ALM index] and total body lean mass). %ALM is suggested to be a better predictor of insulin resistance and diabetes risk than ALM or ALM index.^15,16^

Body fat mass was evaluated as either height-adjusted fat mass index (FMI) or weight-adjusted percentage body fat (% body fat). As the leg region included the entire hip, thigh, and leg, leg fat is referred to as gluteofemoral or lower-body fat. Leg (trunk) fat distribution was assessed by leg (trunk) fat relative to total fat mass and expressed as %LF (%TF). We have shown that %LF (%TF) was related to a favorable (unfavorable) cardiometabolic profile.^26^

Data were presented as mean ± standard deviation unless otherwise stated. Due to deviation from normal distribution, hsCRP and HOMA-IR were logarithmically transformed for analysis. Comparisons between two groups were made with two-sample *t*-test or chi square test when appropriate. Differences among underweight, normal-weight, and overweight women were analyzed using analysis of variance and then Bonferroni's multiple comparison procedure. Overweight women were included as the internal reference. A two-tailed value of *p* < 0.05 was considered significant. Statistics were performed with SPSS system version 23 (SPSS Inc., Chicago, IL).

## Results

Among 307 women, 42 (13.7%), 251 (81.7%), and 14 (4.6%) women were underweight, normal weight, and overweight, respectively (Table 1). The percentage of babies with low birth weight (<2500 g) was low in the total population (2.2%, 5/226).

**Table 1.** Birth Weight and Trajectories of Body Mass Index of Underweight, Normal-Weight, and Overweight Japanese University Females Majoring in Nutrition Sciences

	Underweight	Normal weight	Overweight	#
	***n* = 42**	***n* = 251**	***n* = 14**	
Birth weight <2500 g (*n*, %)	2/32, 6.3	3/182, 1.6	0/12, 0	
Weight (kg) at birth	2991 ± 369	3196 ± 388	3146 ± 306	a
At age 12	37.5 ± 4.9	43.5 ± 6.4	56.2 ± 8.9	a,b,c
At age 15	43.6 ± 4.7	49.5 ± 5.7	62.7 ± 9.9	a,b,c
At age 20	44.4 ± 5.0	51.9 ± 4.9	67.6 ± 6.8	a,b,c
Height (cm) at age 12	148.1 ± 6.3	151.0 ± 6.3	154.9 ± 7.5	c
At age 15	155.9 ± 4.8	157.1 ± 5.0	159.1 ± 5.9	
At age 20	159.1 ± 5.1	158.9 ± 5.0	159.4 ± 5.6	
BMI (kg/m^2^) at age 12	17.1 ± 1.3	19.0 ± 2.1	23.3 ± 2.2	a,b,c
At age 15	18.0 ± 1.7	20.0 ± 1.8	24.7 ± 2.8	a,b,c
At age 20	17.5 ± 1.4	20.6 ± 1.4	26.6 ± 1.6	a,b,c

Mean ± standard deviation.

#: Significantly different at *p* < 0.05 or less. a and b: underweight versus normal weight and overweight, respectively, c: normal weight versus overweight.

BMI, body mass index.

Birth weight was lower in underweight compared to normal-weight women (Table 1). However, the percentage of babies with low birth weight did not differ significantly among the three groups (analysis of variance *p* = 0.229). Weight at age 12, 15, and 20 was also lower in underweight compared with normal-weight women, but height did not differ, and hence BMI at three time points was lower in underweight women.

As shown in Table 2, weight-adjusted lean mass (%TBLM and %ALM) and relative leg fat to total fat mass (%LF) were the highest levels in underweight women, intermediate levels in normal-weight women, and lowest levels in overweight women. In contrast, ALM index, FMI, % body fat, %TF, and trunk/leg fat ratio showed a graded increase, with the lowest levels observed in underweight women, intermediate levels in normal-weight women, and highest levels in overweight women (Table 2).

**Table 2.** Body Composition of Underweight, Normal-Weight, and Overweight Japanese Female Students

	Underweight	Normal weight	Overweight	#
	***n* = 42**	***n* = 251**	***n* = 14**	
Age (years)	20.5 ± 1.2	20.6 ± 1.2	20.7 ± 1.0	
BMI (kg/m^2^)	17.5 ± 1.4	20.6 ± 1.4	26.6 ± 1.6	a,b,c
Waist (cm)	66.4 ± 3.6	71.5 ± 5.0	83.9 ± 4.6	a,b,c
Height (cm)	159.1 ± 5.1	158.9 ± 5.0	159.4 ± 5.6	
Weight (kg)	44.4 ± 5.0	51.9 ± 4.9	67.6 ± 6.8	a,b,c
BLM (kg)	32.1 ± 2.4	34.3 ± 3.0	38.6 ± 2.7	a,b,c
ALM (kg)	13.6 ± 1.3	14.9 ± 1.5	17.2 ± 1.6	a,b,c
%BLM (%)	73.1 ± 8.9	66.2 ± 4.6	57.3 ± 3.6	a,b,c
%ALM (%)	30.8 ± 3.1	28.7 ± 2.1	25.5 ± 2.1	a,b,c
ALM index (kg/m^2^)	5.3 ± 0.4	5.9 ± 0.5	6.8 ± 0.4	a,b,c
Total FM (kg)	9.5 ± 2.4	14.6 ± 3.3	25.0 ± 5.6	a,b,c
%Total FM (%)	21.5 ± 4.4	28.3 ± 4.6	37.4 ± 4.9	a,b,c
FM index (kg/m^2^)	3.7 ± 0.8	5.8 ± 1.3	9.8 ± 1.9	a,b,c
Gluteofemoral/total fat (%)	42.6 ± 3.8	39.0 ± 4.0	35.3 ± 5.3	a,b,c
Trunk/total fat (%)	45.5 ± 3.7	48.3 ± 3.8	52.1 ± 5.1	a,b,c

Mean ± standard deviation.

#: Same as in Table 1.

ALM, appendicular lean mass; BLM, body lean mass; FM, fat mass.

In Pearson's correlation analyses, %ALM showed inverse associations with BMI (*r* = −0.592), FMI (*r* = −0.727), % body fat (*r* = −0.798), and fasting insulin (*r* = −0.185) and HOMA-IR (*r* = −0.198) (all *p* < 0.001). In contrast, ALM index showed no association with fasting insulin and HOMA-IR (*r* = 0.07 and 0.08, *p* = 0.20 and 0.14, respectively) and positive, not inverse, association with BMI (*r* = 0.662), FMI (*r* = 0.347) (both *p* < 0.001), and % body fat (*r* = 0.123, *p* = 0.03).

Among 129 women who underwent OGTT, one underweight and three normal-weight women had IGT, while no overweight women had. There was no difference in the proportion of women with IGT between underweight and normal-weight women (1/22 vs. 3/99, *p* = 0.78). Fasting glucose and insulin and hence HOMA-IR were higher in overweight compared with underweight and normal-weight women, although HOMA-β did not differ (Table 3). Serum leptin showed a stepwise increase, whereas adiponectin showed a stepwise decrease from underweight to overweight women. However, underweight and normal-weight women did not differ in the area under the concentration curve of glucose and insulin, HbA1c, serum lipids, and lipoproteins. There was also no difference in blood hemoglobin and the prevalence of anemia (hemoglobin <12.0 g) among the three groups of women.

**Table 3.** Glucose and Lipid Metabolism of Underweight, Normal-Weight, and Overweight Japanese Female Students Majoring in Nutrition Sciences

	Underweight	Normal weight	Overweight	
	***n* = 42**	***n* = 251**	***n* = 14**	
**OGTT**	***n* = 22**	***n* = 99**	***n* = 8**	
Age (years)	20.5 ± 1.2	20.6 ± 1.2	20.7 ± 1	
Fasting glucose (mg/dL)	83 ± 8	83 ± 7	88 ± 7	b,c
30-minute glucose (mg/dL)	122 ± 26	120 ± 22	120 ± 24	
60-minute glucose (mg/dL)	98 ± 24	104 ± 34	103 ± 37	
120-minute glucose (mg/dL)	94 ± 22	93 ± 23	90 ± 16	
Fasting insulin (μU/mL)	5.8 ± 2.9	6.0 ± 3.3	9.9 ± 5.2	b,c
30-minute insulin (μU/mL)	50 ± 17	52 ± 34	68 ± 46	
60-minute insulin (μU/mL)	42 ± 24	43 ± 25	67 ± 39	b
120-minute insulin (μU/mL)	39 ± 21	40 ± 25	44 ± 26	
AUCg (mg/dL/2 hours)	202 ± 35	205 ± 43	205 ± 45	
AUCi (μU/mL/2 hours)	77 ± 28	79 ± 38	108 ± 51	
HbA1c (%)	5.2 ± 0.2	5.2 ± 0.2	5.2 ± 0.3	
HOMA-IR	1.2 ± 0.7	1.2 ± 0.8	2.6 ± 1.9	b,c
HOMA-β	128 ± 118	123 ± 98	142 ± 71	
Triglyceride (mg/dL)	66 ± 66	56 ± 25	74 ± 29	
Cholesterol (mg/dL)	175 ± 26	183 ± 26	201 ± 48	
HDL cholesterol (mg/dL)	74 ± 14	75 ± 13	75 ± 15	
LDL cholesterol (mg/dL)	88 ± 22	97 ± 23	112 ± 40	c
Leptin (ng/mL)	5.8 ± 2.4	8.6 ± 3.6	15.3 ± 3.5	a,b,c
Adiponectin (mg/L)	12.8 ± 4.2	11.4 ± 4.1	9.1 ± 5.5	b
hsCRP (μg/dL)	21 ± 44	29 ± 72	46 ± 50	
Blood hemoglobin (g/dL)	13.1 ± 1.0	13.0 ± 1.0	13.0 ± 1.3	
Anemia (*n*, %)	4, 9.5	39, 15.6	3, 21.4	

Mean ± standard deviation.

#: Same as in Table 1.

AUCg, area under the concentration curve of glucose; AUCi, area under the concentration curve of insulin; HbA1c, glycated hemoglobin; HDL, high-density lipoprotein; HOMA-IR, homeostasis model assessment of insulin resistance; HOMA-β, homeostasis model assessment of secretion; hsCRP, high-sensitivity C-reactive protein; LDL, low-density lipoprotein; OGTT, oral glucose tolerance test.

Daily dietary intake of energy, carbohydrate, fat, and protein did not differ among the three groups of women (Table 4). However, relative intake of energy and protein per kilogram of body mass was the highest in underweight women, intermediate levels in normal-weight women, and lowest levels in overweight women.

**Table 4.** Daily Dietary Intake in Young Women

	Underweight	Normal weight	Overweight	#
	***n* = 32**	***n* = 166**	***n* = 8**	
Energy (kcal)	1828 ± 489	1958 ± 765	1590 ± 331	
Carbohydrate (g)	241 ± 62	265 ± 116	219 ± 41	
Protein (g)	64 ± 18	65 ± 22	52 ± 12	
Fat (g)	64 ± 24	67 ± 27	53 ± 18	
Carbohydrate (% energy)	53.1 ± 6.3	54.1 ± 5.7	55.5 ± 5	
Protein (% energy)	14 ± 1.8	13.4 ± 1.7	13.2 ± 1.3	
Fat (% energy)	31.1 ± 6.2	30.8 ± 5	29.6 ± 5	
Energy (kcal/kg·BW)	41.5 ± 11.2	37.7 ± 14.1	24.0 ± 6.0	b,c
Protein (g/kg·BW)	1.4 ± 0.4	1.3 ± 0.4	0.8 ± 0.2	a,b,c

Mean ± standard deviation.

#: Same as in Table 1.

BW, body weight.

## Discussion

As far as we know, this study is the first to show that young underweight women had lower weight at birth and age 12, 15, and 20 years. In addition, they had higher relative leg (gluteofemoral) fat to total fat mass (%LF) and weight-adjusted appendicular skeletal muscle mass (%ALM). Glucose tolerance and lipid profile did not differ between underweight and normal-weight women.

Reduced birth weight is a widely used indicator of retarded fetal growth and intrauterine malnutrition. Although the proportion of female infants with birth weight <2500 g has been reported to be increased from 4.8% in 1980 to 10.7% in 2012 in Japan,^27^ fetal malnutrition was not possibly the primary factor in this study since the percentage of infants with birth weight <2500 g was not high in underweight women (6.3%). Blood hemoglobin, anemia prevalence, and daily intake of energy and macronutrients did not differ among the three groups of women, suggesting that our underweight women were unlikely to be undernourished, but healthy.

Although reasons of the increase in thinness in young Japanese women remain unclear, studies found a negative body image and weight loss behaviors among a substantial proportion of normal-weight children and adolescents.^7–9^ This could manifest as a rising thinness prevalence. Indeed, some have suggested that programs to prevent overweight and obesity may actually cause unintended harm.^10,11^ Higher prevalence of underweight in young Japanese women may be, in part, due to unnecessary weight control in nonobese individuals as it has been shown that body dissatisfaction and desire for thinness are commonplace in high socioeconomic status settings across world regions in women in general,^28,29^ and in young Japanese women in particular.^7,30^ It may also be related to eating problems.^31^ Although we did not investigate eating behaviors, women with unusual dietary habits were excluded from the study and daily energy intake did not differ between underweight and normal-weight women.

A previous study suggested that the continuous increase in the smoking rate may be associated with an increase in thinness in Japanese women of childbearing age.^3^ However, this is not the case with our participants because no participant reported a smoking habit.^21^ Taken together, this study has clearly shown that in Japanese female students majoring in nutrition sciences, underweight is constitutional and is not due to calorie restriction. Although reasons why some Japanese female students majoring in nutrition sciences are underweight are not clear, a recent genome-wide association study identifies anaplastic lymphoma kinase as a thinness gene, which is involved in the resistance to weight gain.^32^

Skeletal muscle is a major site of insulin-mediated glucose disposal in the postprandial state.^13^ Disruption of glucose uptake by muscle directly contributes to the development of insulin resistance.^33^ In addition, in the fasting state, smooth muscle is a site of clearance of triglyceride in very low-density lipoprotein particles and production of HDL particles.^34^ Despite lower absolute ALM and ALM index in underweight women, glucose disposal rate during OGTT (area under the concentration curve of glucose) and lipid metabolism did not differ between underweight and normal-weight young women as previously reported.^35^ This may be explained, in part, by higher %LF associated with lower %TF and higher %ALM in underweight women.

We have shown that %LF (%TF) was related to a favorable (unfavorable) cardiometabolic profile.^26^ An inverse association of relative muscle mass (%ALM) with fasting insulin and HOMA-IR was confirmed even in young Japanese women in this study as previously reported in elderly populations.^15,16^

Studies are limited on glucose tolerance in underweight and normal-weight people. We found two studies from the same Japanese laboratory.^35,36^ The former study found that although IGT was more prevalent in postmenopausal compared with young underweight women, glucose tolerance did not differ between 31 young underweight and 13 normal-weight women.^35^ The latter study found that IGT was more prevalent in 98 young underweight compared with 56 normal-weight women (13.3% vs. 1.8%).^36^

Although lower insulin secretion, lower muscle function, insulin resistance in muscle, and adipose tissue may contribute to elevated postload glucose values,^35,36^ a low carbohydrate intake before oral glucose testing is another forgotten factor, which is associated with elevated postload glucose values.^37^ As young underweight women in the latter study were underweight probably due to desire to be thin,^36^ young underweight women with IGT consumed, on average, 134 g of carbohydrate a day (46.7% of 1152 kcal), which is lower than the recommended daily carbohydrate intake (>150 g).^37^ In contrast, four underweight women with IGT in this study consumed, on average, 251 g a day (204–273 g).

Some studies^38–40^ examined the weight trajectory from birth in relation to type 2 diabetes, but not to underweight in young adulthood. As far as we know, this study may be the first to investigate weight trajectories since birth in underweight young women.

The strengths of this study include homogeneous study population with scarce confounding factors,^19–21^ and accurate and reliable measures of body composition by DXA, which provided direct measures of fat mass and skeletal muscle mass and their distribution. Several limitations of this study include the cross-sectional design, relatively small sample size, and a single measurement of biochemical variables. Students majoring in nutrition sciences may be more health conscious and diet aware. In addition, as the participation was voluntary, female students who pay more attention to health may be more likely to participate. Therefore, participants in this study may not be representative of the overall population of young healthy women in Japan. We used crude measures of insulin resistance/sensitivity, which may be less accurate. Menstrual cycles were not adjusted, and statistical power was not calculated. As we studied Japanese only, results may not be generalized to other races or ethnicities.

## Conclusions

Some Japanese female students are underweight not because of a strong desire for thinness, but rather because they were born lighter and remained lean until young adults, that is, constitutionally thin. Underweight was associated with higher relative leg fat to total fat mass (%LF) and higher relative muscle mass, both of which may be related to glucose and lipid metabolism indistinguishable from normal-weight women.

## Abbreviations Used

ALM: appendicular lean mass
AUCg: area under the concentration curve of glucose
AUCi: area under the concentration curve of insulin
BLM: body lean mass
BMI: body mass index
BW: body weight
CV: coefficient of variation
DXA: dual-energy X-ray absorptiometry
FM: fat mass
FMI: fat mass index
HbA1c: glycated hemoglobin
HDL: high-density lipoprotein
HOMA-IR: homeostasis model assessment of insulin resistance
HOMA-β: homeostasis model assessment of secretion
hsCRP: high-sensitivity C-reactive protein
IGT: impaired glucose tolerance
LDL: low-density lipoprotein
OGTT: oral glucose tolerance test

## References

[1] The National Health and Nutrition Survey. Ministry of Health, Labour and Welfare. 2016. Available at: www.nibiohn.go.jp/eiken/english/research/project_nhns.html Accessed September 1, 2020.

[2] Funatogawa I, Funatogawa T, Nakao M, Karita K, Yano E. Changes in body mass index by birth cohort in Japanese adults: Results from the National Nutrition Survey of Japan 1956–2005. Int J Epidemiol 2009;38:83–92. 18782894 10.1093/ije/dyn182 PMC2639362

[3] Takimoto H, Yoshiike N, Kaneda F, Yoshita K. Thinness among young Japanese women. Am J Public Health 2004;94:1592–1595. 15333320 10.2105/ajph.94.9.1592 PMC1448499

[4] Sugawara A, Saito K, Sato M, Kodama S, Sone H. Thinness in Japanese young women. Epidemiology 2009;20:464–465. 19363358 10.1097/EDE.0b013e31819ed4ed

[5] Brown WJ, Mishra G, Kenardy J, Dobson A. Relationship between body mass index and well-being in young Australian women. Int J Obes Relat Metab Disord 2000;24:1360–1368. 11093300 10.1038/sj.ijo.0801384

[6] Kriaucioniene V, Raskiliene A, Petrauskas D, Petkeviciene J. Trends in eating habits and body weight status, perception patterns and management practices among first-year students of Kaunas (Lithuania) Universities, 2000–2017. Nutrients 2021;13:1599. 34064684 10.3390/nu13051599 PMC8151775

[7] Kaneko K, Kiriike N, Ikenaga K, Miyawaki D, Yamagami S. Weight and shape concerns and dieting behaviors among pre-adolescents and adolescents in Japan. Psychiatry Clin Neurosci 1999;53:365–371. 10459738 10.1046/j.1440-1819.1999.00559.x

[8] Rozin P, Fallon A. Body image, attitudes to weight, and misperceptions of figure preferences of the opposite sex: A comparison of men and women in two generations. J Abnorm Psychol 1988;97:342–345. 3192829 10.1037//0021-843x.97.3.342

[9] Bun CJ, Schwiebbe L, Schütz FN, Bijlsma-Schlösser JF, Hirasing RA. Negative body image and weight loss behaviour in Dutch school children. Eur J Public Health 2012;22:130–133. 21441558 10.1093/eurpub/ckr027

[10] Carter FA, Bulik CM. Childhood obesity prevention programs: How do they affect eating pathology and other psychological measures? Psychosom Med 2008;70:363–371. 18378876 10.1097/PSY.0b013e318164f911

[11] O'Dea JA. Prevention of child obesity: ‘First, do no harm’. Health Educ Res 2005;20:259–265. 15328303 10.1093/her/cyg116

[12] Li JB, Wu Y, Gu D, Li H, Zhang X. Prevalence and temporal trends of presarcopenia metrics and related body composition measurements from the 1999 to 2006 NHANES. BMJ Open 2020;10:e034495. 10.1136/bmjopen-2019-034495 PMC7410000 32759238

[13] Bonadonna RC, Saccomani MP, Seely L, et al. Glucose transport in human skeletal muscle. The in vivo response to insulin. Diabetes 1993;42:191–198. 8093605 10.2337/diab.42.1.191

[14] Chen LK, Liu LK, Woo J, et al. Sarcopenia in Asia: Consensus report of the Asian Working Group for Sarcopenia. J Am Med Dir Assoc 2014;15:95–101. 24461239 10.1016/j.jamda.2013.11.025

[15] Bijlsma AY, Meskers CG, van Heemst D, Westendorp RG, de Craen AJ, Maier AB. Diagnostic criteria for sarcopenia relate differently to insulin resistance. Age (Dordr) 2013;35:2367–2375. 23407994 10.1007/s11357-013-9516-0 PMC3824998

[16] Lee SW, Youm Y, Lee WJ, et al. Appendicular skeletal muscle mass and insulin resistance in an elderly Korean population: The Korean social life, health and aging project-health examination cohort. Diabetes Metab J 2015;39:37–45. 25729711 10.4093/dmj.2015.39.1.37 PMC4342535

[17] Moon SS. Low skeletal muscle mass is associated with insulin resistance, diabetes, and metabolic syndrome in the Korean population: The Korea National Health and Nutrition Examination Survey (KNHANES) 2009–2010. Endocr J 2014;61:61–70. 24088600 10.1507/endocrj.ej13-0244

[18] Son JW, Lee SS, Kim SR, et al. Low muscle mass and risk of type 2 diabetes in middle-aged and older adults: Findings from the KoGES. Diabetologia 2017;60:865–872. 28102434 10.1007/s00125-016-4196-9

[19] Kitaoka K, Takeuchi M, Tsuboi A, et al. Increased adipose and muscle insulin sensitivity without changes in serum adiponectin in young female collegiate athletes. Metab Syndr Relat Disord 2017;15:246–251. 28318384 10.1089/met.2017.0011 PMC5485219

[20] Tanaka S, Wu B, Honda et al. Associations of lower-body fat mass with favorable profile of lipoproteins and adipokines in healthy, slim women in early adulthood. J Atheroscler Thromb 2011;18:365–372. 21233589 10.5551/jat.7229

[21] Tanaka M, Yoshida T, Bin W, Fukuo K, Kazumi T. FTO, abdominal adiposity, fasting hyperglycemia associated with elevated HbA1c in Japanese middle-aged women. J Atheroscler Thromb 2012;19:633–642. 22504289 10.5551/jat.11940

[22] Wu B, Huang J, Zhang L, et al. An integrative approach to investigate the association among high-sensitive C-reactive protein, body fat mass distribution, and other cardiometabolic risk factors in young healthy women. Methods 2018;145:60–66. 29702223 10.1016/j.ymeth.2018.04.016 PMC6064666

[23] Okubo H, Sasaki S, Rafamantanantsoa HH, Ishikawa-Takata K, Okazaki H, Tabata I. Validation of self-reported energy intake by a self-administered diet history questionnaire using the doubly labeled water method in 140 Japanese adults. Eur J Clin Nutr 2008;62:1343–1350. 17671444 10.1038/sj.ejcn.1602858

[24] American Diabetes Association. 2. Classification and Diagnosis of Diabetes: Standards of Medical Care in Diabetes-2021. Diabetes Care 2021;44(Suppl. 1):S15–S33. 33298413 10.2337/dc21-S002

[25] Wallace TM, Levy JC, Matthews DR. Use and abuse of HOMA modeling. Diabetes Care 2004:27:1487–1495. 15161807 10.2337/diacare.27.6.1487

[26] Wu B, Huang J, Fukuo K, Suzuki K, Yoshino G, Kazumi T. Different associations of trunk and lower-body fat mass distribution with cardiometabolic risk factors between healthy middle-aged men and women. Int J Endocrinol 2018;2018:1289485. 29531527 10.1155/2018/1289485 PMC5817354

[27] Vital Statistics of Japan, vol. 2013, Health, Labour and Welfare Statistics Association, Tokyo, 2012. Available at: https://www.mhlw.go.jp/english/database/db-hw/vs01.html” Ministry of Health, Labour and Welfare (mhlw.go.jp).

[28] Swami V, Frederick DA, Aavik T, et al. The attractive female body weight and female body dissatisfaction in 26 countries across 10 world regions: Results of the international body project I. Pers Soc Psychol Bull 2010;36:309–325. 20179313 10.1177/0146167209359702

[29] Wardle J, Haase AM, Steptoe A. Body image and weight control in young adults: International comparisons in university students from 22 countries. Int J Obes (Lond) 2006;30:644–651. 16151414 10.1038/sj.ijo.0803050

[30] Hayashi F, Takimoto H, Yoshita K, Yoshiike N. Perceived body size and desire for thinness of young Japanese women: A population-based survey. Br J Nutr 2006;96:1154–1162. 17181892 10.1017/bjn20061921

[31] Mase T, Miyawaki C, Kouda K, Fujita Y, Ohara K, Nakamura H. Relationship of a desire of thinness and eating behavior among Japanese underweight female students. Eat Weight Disord 2013;18:125–132. 23760840 10.1007/s40519-013-0019-x

[32] Orthofer M, Valsesia A, Mägi R, et al. Identification of ALK in thinness. Cell 2020;181:1246.e22–1262.e22. 32442405 10.1016/j.cell.2020.04.034

[33] Wolfe RR. The underappreciated role of muscle in health and disease. Am J Clin Nutr 2006;84:475–482. 16960159 10.1093/ajcn/84.3.475

[34] Kiens B, Lithell H. Lipoprotein metabolism influenced by training-induced changes in human skeletal muscle. J Clin Invest 1989;83:558–564. 2643634 10.1172/JCI113918 PMC303715

[35] Someya Y, Tamura Y, Suzuki R, et al. Characteristics of glucose metabolism in underweight Japanese women. J Endocr Soc 2018;2:279–289. 29600294 10.1210/js.2017-00418 PMC5838825

[36] Sato M, Tamura Y, Nakagata T, et al. Prevalence and features of impaired glucose tolerance in young underweight Japanese women. J Clin Endocrinol Metab 2021;106:e2053–e2062. 33512496 10.1210/clinem/dgab052

[37] Klein KR, Walker CP, McFerren AL, Huffman H, Frohlich F, Buse JB. Carbohydrate intake prior to oral glucose tolerance testing. J Endocr Soc 2021;5:bvab049. 33928207 10.1210/jendso/bvab049 PMC8059359

[38] Katanoda K, Noda M, Goto A, Mizunuma H, Lee JS, Hayashi K. Being underweight in adolescence is independently associated with adult-onset diabetes among women: The Japan Nurses' Health Study. J Diabetes Investig 2019;10:827–836. 10.1111/jdi.12947 PMC6497776 30290067

[39] Slining MM, Kuzawa CW, Mayer-Davis EJ, Adair LS. Evaluating the indirect effect of infant weight velocity on insulin resistance in young adulthood: A birth cohort study from the Philippines. Am J Epidemiol 2011;173:640–648. 21317221 10.1093/aje/kwq435 PMC3105264

[40] Eriksson JG, Kajantie E, Lampl M, Osmond C. Trajectories of body mass index amongst children who develop type 2 diabetes as adults. J Intern Med 2015;278:219–226. 25683182 10.1111/joim.12354

